# *In Vitro* Praziquantel Test Capable of Detecting Reduced *In Vivo* Efficacy in *Schistosoma mansoni* Human Infections

**DOI:** 10.4269/ajtmh.2010.10-0413

**Published:** 2010-12-06

**Authors:** Poppy H. L. Lamberton, Sarah C. Hogan, Narcis B. Kabatereine, Alan Fenwick, Joanne P. Webster

**Affiliations:** Department of Infectious Disease Epidemiology, Faculty of Medicine, Imperial College London, London, United Kingdom; Vector Control Division, Ministry of Health, Kampala, Uganda

## Abstract

Although great reductions in human schistosomiasis have been observed after praziquantel (PZQ) mass drug administration (MDA), some individuals remain infected after multiple treatments. Many MDA programs now require monitoring for drug efficacy as a key component. No molecular tools for PZQ resistance currently exist and investigations into the dose of PZQ required to kill 50% of adult worms *in vivo* (ED_50_) present ethical, logistical, and temporal restraints. We, therefore, assessed the feasibility and accuracy of a rapid, inexpensive *in vitro* PZQ test in the laboratory and directly in the field in Uganda under MDA in conjunction with highly detailed infection intensity, clearance, and reinfection data. This test strongly differentiated between subsequently cleared and uncleared infections as well as differences between parasite populations pre- and post-PZQ treatments, advocating its use for on-the-spot monitoring of PZQ efficacy in natural foci. After only a few treatments, uncleared parasites were identified to be phenotypically different from drug-sensitive parasites, emphasizing the urgent need for monitoring of these repeatedly PZQ-treated populations.

## Introduction

Praziquantel (PZQ) is the only current drug of choice for the treatment of schistosomiasis, with the 54th World Health Assembly setting a target of treating 75% of school-age children in high endemic regions by the end of 2010.^1^ The Ugandan National Government, with assistance from the Schistosomiasis Control Initiative (SCI), has administered mass PZQ treatment over 7 years, with infection prevalences greatly reduced from 33.4–49.3% to 9.7–29.6% and intensities reduced from 105.7–386.8 eggs per gram (epg) of feces to 11.6–84.1 epg in the first 2 years alone.^2^,^3^ Analyses of qualitative data from the control program within Uganda are also reporting an increased demand for continued treatment, likely to lead to further widespread PZQ use.^4^ There are, as of yet, no clear reports of PZQ resistance, including in China with over 20 years of treatment; there were early concerns of resistance in Egypt,^5^ but resistance was not observed to have spread with continued mass drug administration (MDA).^6^ However, the relatively new strong MDA selective pressures now occurring in sub-Saharan Africa are acting without the high refugia found in *Schistosoma japonicum* populations or the additional constraining pressures of intense mollusciding as has been implemented for over 50 years in both Egypt and China,^7^,^8^ both of which could dilute and/or limit any potential adaptation and establishment of PZQ-resistant genotypes. Crucially, MDA on *Schistosoma mansoni* populations in sub-Saharan Africa has very recently been shown to impact the parasite population diversity,^9^ which could indicate early changes, such as potential development, and spread of any changes in PZQ efficacy, like resistance; this is particularly because schistosomes have sufficient genetic variability to evolve^10^–^12^ and coevolve^13^–^15^ and artificial selection for PZQ resistance has been shown within the laboratory in as few as six generations.^16^

At present, it is possible to test for PZQ resistance using investigations into the dose of PZQ required to kill 50% of adult worms *in vivo* (ED_50_) or *in vitro* PZQ assays on adult worms derived from passage through laboratory rodents,^17^,^18^ with similar *in vitro* assays also used to test the comparative effectiveness of other drugs.^19^,^20^ These assays, however, present strong ethical, logistical, and biological disadvantages, requiring high laboratory animal usage and several months for passage, with the significant risk of genetic bottlenecks, thereby confounding results obtained from such methodologies.^21^ Because of the location of schistosomes within the mesenteric arteries of their mammalian hosts, direct analysis of adult worms from human infections is not possible, and although novel techniques for molecular assessment on larval stages have been shown,^21^ there are, at present, no molecular markers for PZQ resistance. Low budget, rapid methods for monitoring parasite phenotypes are, therefore, crucial, and they could provide logistically important information concerning the potential PZQ resistance status of natural parasite populations and help answer some of the questions posed about drug resistance in human infections.^22^ The methods presented here, therefore, focus on isolates directly from the field to accurately represent the parasite populations using low-cost, rapid techniques.

Previous work on adult worms, miracidia (larvae hatched from eggs excreted by infected mammalian definitive hosts), cercariae (larvae released from infective molluscan intermediate hosts), and eggs has shown that changes in morphology of *S. mansoni*, when exposed to *in vitro* PZQ, could reflect *in vivo* susceptibility,^23^ with the larval stages highlighted as more sensitive than adult worms.^23^,^24^ Indeed, miracidia (and cercariae) provide an ideal opportunity to monitor putative changes after chemotherapeutic treatment, enabling comparisons between parasite populations without possible biases of clearance trends, infection intensities, human host immune status, and/or PZQ pharmacokinetics. Miracidial shape changes after *in vitro* PZQ exposure were termed tadpole and dumbbell by Liang and others,^24^ with greater percentages of change observed in PZQ-susceptible than PZQ-resistant isolates.^24^ Such tests also showed that a higher proportion of miracidia from susceptible isolates was inactive after 5 minutes of PZQ stress than from resistant isolates.^24^ *In vitro* tests of *S. japonicum* cercariae, miracidia, and eggs have indicated that they are more sensitive to PZQ than *S. mansoni*, potentially explaining the lack of evidence for resistance in *S. japonicum* from China and highlighting the need to monitor *S. mansoni* populations more closely.^25^

Many researchers have pinpointed the need to monitor schistosomes in the field as MDA continues, with techniques to investigate the mode of action of PZQ as well as to monitor PZQ susceptibility in human infections being a necessity for the continued success of such programs.^26^ The laboratory aspect of the current study, therefore, expands on work by Liang and others^24^ by characterizing here the impact of *in vitro* PZQ on resistant, susceptible, and coinfected *S. mansoni* lines under *in vivo* PZQ selection using video capture analysis for detailed observations of miracidia behavior and morphology over time. The morphological aspects of these original tests on *S. mansoni* have, to date, only been evaluated in the field in China on *S. japonicum* from infected water buffalo and goats.^27^ The objective of our study was, therefore, to assess, for the first time to the authors' knowledge, the feasibility of use of this rapid, inexpensive diagnostic test using *in vitro* PZQ directly on *S. mansoni* miracidia from endemic human infections to detect PZQ susceptibility under mass chemotherapeutic pressure in a real field environment. Our measures were uniquely quantified using highly detailed longitudinal infection intensity, clearance, and reinfection data, including repeated sampling from the same children over time.

It was predicted that, when exposed to *in vitro* PZQ, resistant miracidia from the laboratory would show lower proportions of immobility and shape change than the coinfected and susceptible lines, supporting the work of Liang and others^24^ When PZQ treatment was administered *in vivo*, these proportions of inactive and dumbbell-shaped miracidia were predicted to be reduced. It was predicted that children whose infections were cleared by a subsequent PZQ treatment would show greater miracidial inactivity and shape change than individuals whose infections were not completely cleared by a standard PZQ treatment.

## Materials and Methods

### Laboratory.

#### Parasite and host lines.

Three *S. mansoni* lines, PZQ-resistant (R), PZQ-susceptible (S), and coinfection (RS), were used. The R line (EE2) is from an *S. mansoni* isolate established in 1996 from eggs excreted by an inhabitant of the Nile region in Egypt before three non-curative PZQ treatments of 40, 40, and 60 mg/kg, respectively.^28^ The resultant parasite line has an ED_50_ > 100 mg/kg PZQ and was tested at three laboratories in Rome, Bangor, and Egypt.^17^ The S line (MOC) is from an *S. mansoni* isolate established in 1996 from eggs excreted by an inhabitant of the Nile region in Egypt before receiving a single curative 40 mg/kg PZQ treatment.^28^ The resultant parasite line has an ED_50_ < 100 mg/kg PZQ.^17^ Laboratory lines of *Biomphalaria glabrata* and *B. alexandrina* were used to passage the R and S lines before the investigation.

#### Experimental design.

Two groups of four female TO Harlan adult mice (Harlan Olac UK Ltd., UK) were exposed to 220 cercariae of each of one of the three *S. mansoni* lines, R, S, or RS (110 cercariae of each R and S), through paddling in 100 mL of infected water for 30 minutes, giving a total of six groups of four mice. Six weeks post-exposure, all mice were weighed and ear-marked; then, one group of each parasite line received, by oral gavage, a subcurative dose of 50 mg/kg PZQ, and the other group received a water (control) dose. Mice were euthanized 49–56 days post-parasite exposure to achieve maximum parasite return before onset of schistosome-induced morbidity.

Miracidia were hatched from eggs obtained from the livers and spleens of individual mice, which were macerated through a sieve in 250 mL of 0.85% saline. The suspension was left for 10 minutes of sedimentation; then, 240 mL of supernatant were removed, fresh saline was added, and sedimentation and supernatant removal were repeated. The remaining 10 mL of sediment were washed out with 70 mL of spring water and illuminated for 1 hour. Miracidia were pooled within groups, and from each group, nine sets of six miracidia were placed on a Petri dish in 0.1 mL droplets of bottled spring water. Video recordings were taken with an Olympus SZ410 (GX Optical, Suffolk, UK) triocular dissecting microscope attached to a JVC IC-1280E color video camera for 7 minutes. After 2 minutes of recording, 0.5 μL of water or PZQ were added, resulting in 0, 1 × 10^−6^, or 2 × 10^−6^ M PZQ. Three replicates of six new miracidia were recorded per experimental group. The behavior and shape of five miracidia per droplet were recorded continuously for 7 minutes in 2-second intervals using an electronic pacer (Oregon Scientific, UK Ltd., Maidenhead, Berkshire, UK). Viewing order was randomized to prevent observational bias. Miracidial shape was recorded as normal, tadpole, or dumbbell, and the time of any changes was noted.

#### Statistical analysis.

Statistical analyses were performed using SPSS (SPSS, Inc., Chicago, IL). The data for time from PZQ addition until miracidial inactivity and the proportion that became inactive were normally distributed, whereas the time for miracidia to become dumbbell was log-transformed to a normal distribution. The impact of parasite genotype and *in vivo* and *in vitro* PZQ on the time taken to become dumbbell and/or inactive was analyzed using a general linear model. The categorical data of the numbers that became inactive or dumbbell was analyzed using the Kruskal-Wallis test.

### Field.

Individuals were recruited from three primary schools in Mayuge, Uganda: Bugoto Lake View (LV), Bwondha, and Musubi Church of God (CoG). Cohorts consisted of approximately 18 randomly selected children per 6-, 7-, 8-, and 11-year-old age group, with an equal sex ratio within each group. All children aged 7 to 11 years from Bugoto LV and Bwondha had previously received PZQ treatment in July 2003 and July 2004 as part of the SCI annual control program, whereas all children from Musubi CoG and the new 6-year-old recruits from Bugoto LV and Bwondha had received PZQ treatment in July 2004. In July 2004, infection intensity data using 3 successive days of double Kato Katz (KK) smears^29^ were collected pre-treatment, 1 week post-PZQ treatment, and 4 weeks post-PZQ treatment. All children were treated after the pre-treatment sampling point, and all children with positive KK results were retreated at 1 week and 4 weeks post-treatment.

This study was carried out in February 2005, 6 months after the children's most recent exposure to PZQ. Infection intensity was measured as noted above and miracidia hatched from infected individuals' stools (pre-retreatment). All children surveyed were treated with 40 mg/kg PZQ. One week later, the schools were revisited, and intensity measures and hatching was repeated (post-retreatment). After this 1-week follow-up, only children with KK-positive smears were retreated with 40 mg/kg PZQ. Schools were revisited 6 months later (August 2005). Infection intensity and prevalences were measured to investigate clearance and reinfection trends, and all children surveyed were retreated with 40 mg/kg PZQ.

At the pre- and post-retreatment time points, schistosome eggs from individual stool samples were hatched. Samples were passed though a 425-μm sieve with ~1 L of bottled spring water; the suspension was then filtered through a pitchford funnel, consisting of an inner mesh of 80 μm to collect larger debris but allow parasite eggs through it and an outer mesh of 40 μm to collect the eggs. The sediment was rinsed using 1–2 L spring water. The eggs were released into a Petri dish and placed in indirect sunlight for up to 6 hours.

#### PZQ behavior response profiling.

Eight miracidia from each stool sample were placed into each of three wells with 1 mL spring water. Miracidia were observed continuously for 10 seconds every 30 seconds over 7 minutes, with 5 μL water or PZQ solution added after 2 minutes to make concentrations of 0, 1 × 10^−6^, or 2 × 10^−6^ M PZQ. Individual miracidium shapes were recorded as normal, tadpole, or dumbbell at each point of activity across the well (1 cm diameter) and at the end of 7 minutes. Each experiment was repeated three times per child for each *in vitro* PZQ concentration.

#### Statistical analyses.

The percentages of inactive miracidia and shape change after 5 minutes of exposure were analyzed using paired and unpaired *t* tests for comparisons between pre- and post-retreatment, clearance trends, *S. mansoni* prevalence at 4 weeks, *S. mansoni* prevalence at 6 months, recruitment year, and *in vitro* PZQ treatments. A one-way analysis of variance (ANOVA) was used to analyze the effect of the number of previous PZQ treatments. Data that showed a slight positive skew were square root-transformed. Where data could not be transformed to an approximately normal distribution, Kruskal–Wallis or Mann–Whitney tests were used.

### Ethical clearance.

The animal work was performed under Home Office Project License PPL 30/2032, and all procedures were classed as mild. Ethical approval for the parasite material and infection intensity data obtained from the field was obtained from the Uganda National Council of Science and Technology and the Imperial College Research Ethics Committee (ICREC), Imperial College, London, United Kingdom in combination with the ongoing SCI activities. Written consent for the schoolchildren to participate in longitudinal monitoring of the national control program for schistosomiasis and soil transmitted helminths was given by head teachers because of the fact that, in African schools, written consent of the child's guardian is very difficult to obtain (owing to the associated impoverished conditions and often low literacy). The parents/guardians verbal consent was recorded at school committees comprising of parents, teachers, and community leaders after they received satisfactory information about the study. Each individual child also gave verbal consent before recruitment.

## Results

### Laboratory.

**Figure 1.**
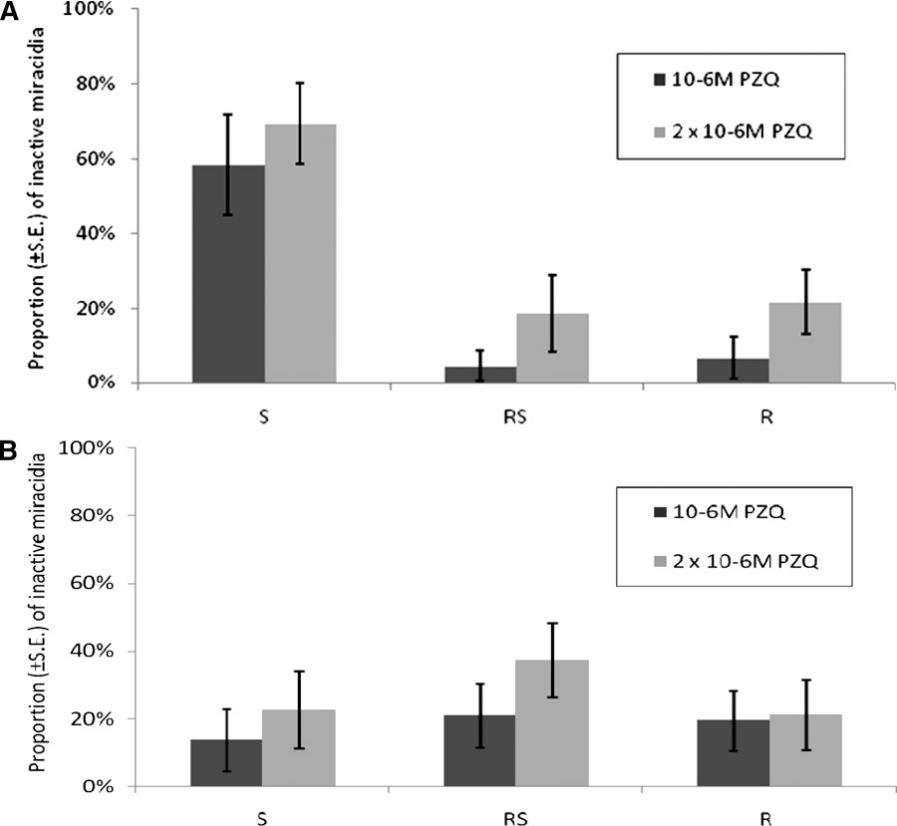
Effect of *in vitro* praziquantel (PZQ) on miracidia from treated and untreated mice. Proportion (±SE) of inactive miracidia PZQ-resistant (R), PZQ-susceptible (S), and co-infected (RS) after 5 minutes exposure to two *in vitro* PZQ concentrations of (**A**) miracidia hatched from untreated mice and (**B**) miracidia hatched from mice treated with 50 mg/kg PZQ.

In the control 0 M PZQ groups (not exposed to *in vitro* PZQ but from treated and untreated mice), all miracidia remained active and normally shaped over the duration of the study. In the *in vitro* PZQ-treated groups, miracidia from the untreated mice showed greater immobility in the higher *in vitro* PZQ concentration than miracidia from treated mice (Kruskal-Wallis test statistic [H] = 32.425, degrees of freedom [df] = 2, *P* < 0.001), with the S miracidia showing higher immobility than both the RS and R (H = 26.418, df = 2, *P* < 0.001) (Figure 1A). Miracidia from PZQ-treated mice showed higher immobility at the higher *in vitro* PZQ concentration (H = 32.685, df = 2, *P* < 0.001), but the proportions of immobile miracidia were not affected by parasite genotype, with the proportion of inactive S miracidia similar to RS and R (Figure 1B). The time taken for miracidia to become inactive after exposure to *in vitro* PZQ was also shortest for S (F_2,26_ = 3.358, *P* = 0.050; S = 46.17 ± 17.01 seconds, RS = 208.86 ± 29.63 seconds, R = 145.20 ± 34.61 seconds).

Alteration to dumbbell shape in all treatment groups occurred faster in 2 × 10^−6^ M (28.16 ± 2.73 seconds) than 1 × 10^−6^ M PZQ (37.01 ± 3.36 seconds; F_1,160_ = 8.039, *P* = 0.005), and miracidia hatched from treated mice took longer to become dumbbell than those from untreated mice (34.56 ± 2.95 seconds and 30.72 ± 3.24 seconds, respectively; F_1,160_ = 4.574, *P* = 0.034).

### Field.

In the control 0 M PZQ groups, all miracidia remained active, and 99.7% were normally shaped throughout the duration of the study. In contrast, shape change and immobility was observed in all *in vitro* PZQ groups. Significantly more miracidia became dumbbell-shaped at the stronger *in vitro* PZQ concentration (F_2,30_ = 24.237, *P* < 0.001), and more miracidia were inactive after 5 minutes at the higher concentration both pre- (F_2,21_ = 21.999, *P* < 0.001) and post-treatment (F_2,18_ = 7.103, *P* = 0.005) than at the lower 10^−6^-M PZQ concentration.

#### Impact of human chemotherapy.

**Figure 2.**
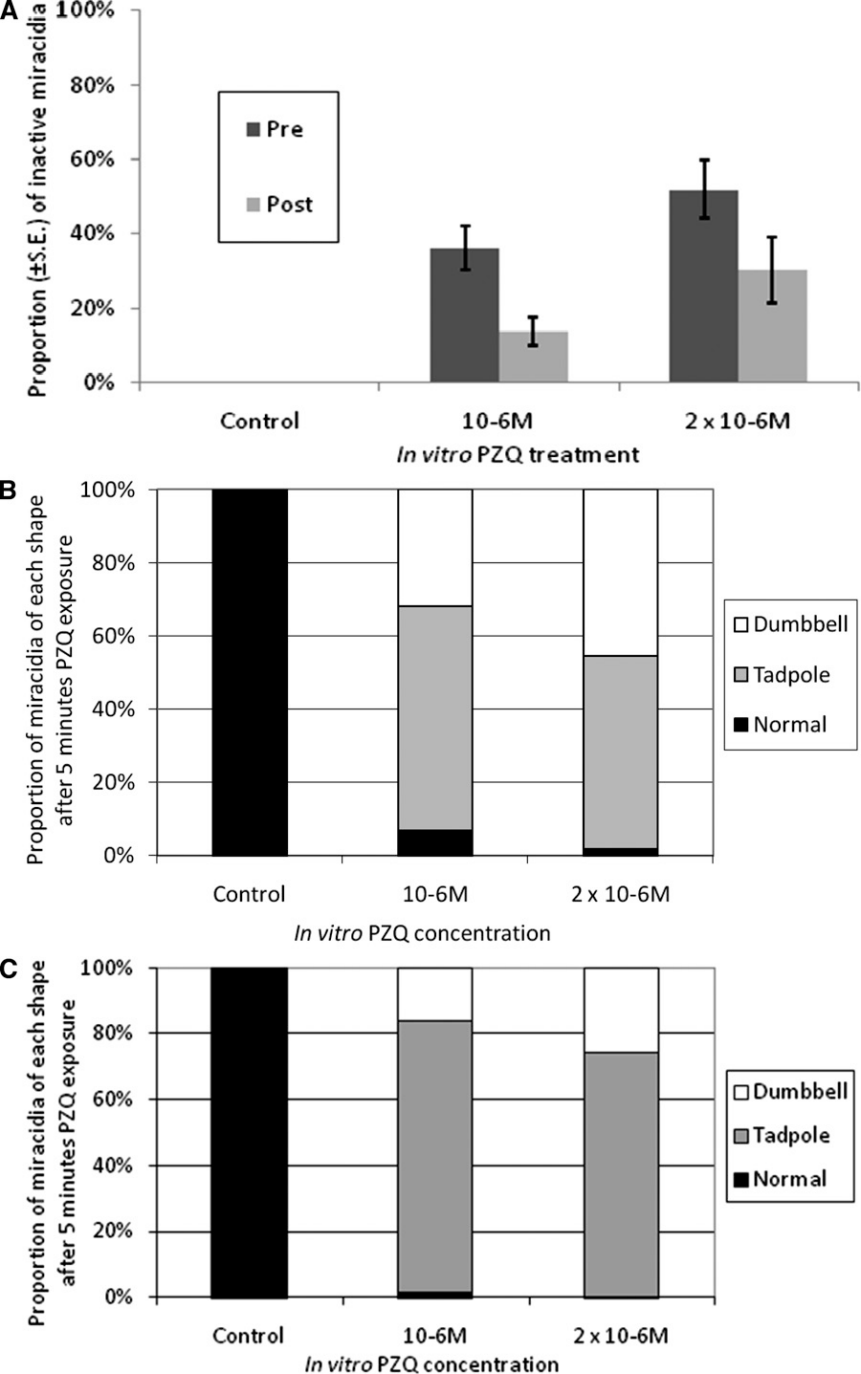
Differences between parasite populations pre- and post-praziquantel (PZQ) treatment. (**A**) Percentage of inactive miracidia per treatment group (±SE) after 5 minutes exposure to *in vitro* PZQ treatment of miracidia hatched at two different time points. One group hatched pre-retreatment with PZQ (pre), and the other group hatched from individuals 1 week after retreatment with 40 mg/kg PZQ (post). Proportion of miracidia that was normal-, tadpole-, or dumbbell-shaped after 5 minutes exposure to *in vitro* PZQ treatment (**B**) pre-retreatment and (**C**) 1 week post-retreatment.

**Figure 3.**
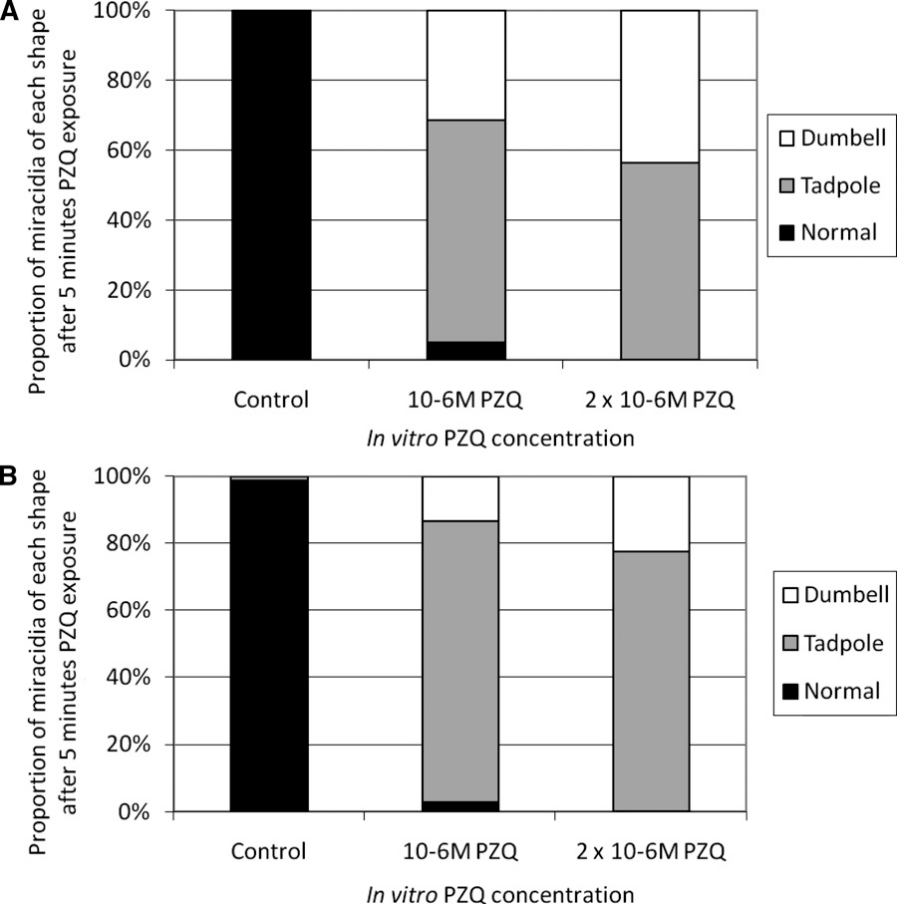
Variations in clearance rates represented by *in vitro* tests. Proportion of miracidia hatched pre-retreatment, which were normal-, tadpole-, or dumbbell-shaped after 5 minutes exposure to the *in vitro* treatment, from individuals whose infection intensities were (**A**) lower 1 week post-retreatment or (**B**) higher 1 week post-retreatment.

The proportion of inactive (paired *t* test, *t* = 2.709, df = 7, *P* = 0.030) (Figure 2A) and dumbbell (paired: *t* = 2.538, df = 7, *P* = 0.039; unpaired: *t* = 2.208, df = 12, *P* = 0.047) (Figure 2B and C) miracidia 5 minutes after *in vitro* PZQ exposure was significantly lower 1 week post-retreatment than in those hatched pre-retreatment. In addition, however, the number of normally shaped miracidia was higher pre-retreatment than post-retreatment in both PZQ concentrations, although only nearly statistically significant in the paired samples (paired: *t* = 1.915, df = 7, *P* = 0.097; unpaired: *t* = 1.367, df = 12, *P* = 0.197). Of miracidia hatched from eight children pre-retreatment, four children had lower infection intensities 1 week post-retreatment, whereas four had higher epgs. Of those with lower infection intensities (lower epgs), there was a non-significant trend for a greater proportion of dumbbell miracidia pre-treatment than in those that were not lowered by MDA, whereas the number of normal-shaped miracidia remained unaffected (Figure 3A and B). Miracidia hatched from individuals who were KK-negative at 4 weeks (5 months before this study) and therefore, assumed-to-be-new PZQ-naive infections showed higher inactivity than that from individuals who had still been KK-positive at 4 weeks (even after two or more PZQ treatments).

#### *S. mansoni* prevalence at follow-up.

**Figure 4.**
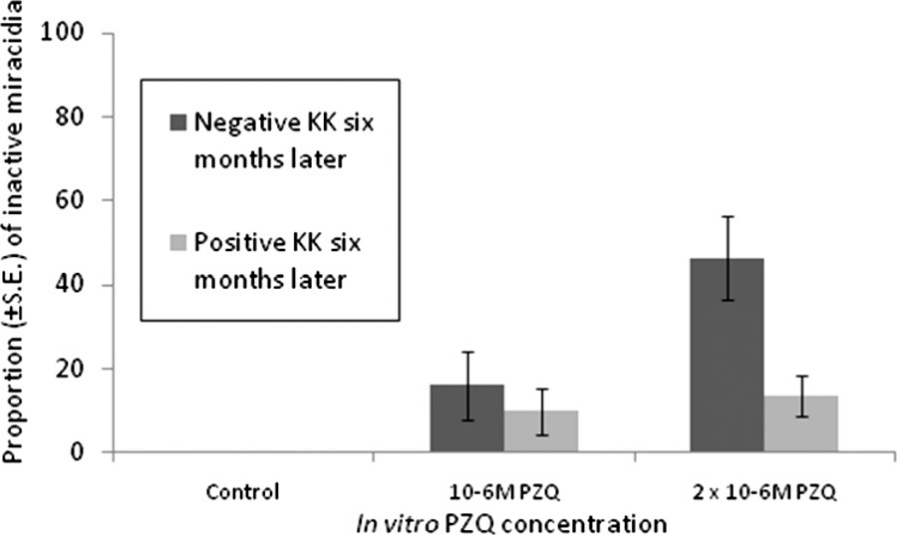
Variations in miracidia activity correlated with long-term parasite clearance. Percentage of inactive miracidia (±SE), hatched from individuals 1 week post-retreatment with 40 mg/kg praziquantel (PZQ), after 5 minutes exposure to *in vitro* PZQ treatment that was either Kato Katz-negative 6 months later, meaning that the infection had been cleared by a single subsequent PZQ treatment, or Kato Katz-positive 6 months later and therefore, with potentially reduced susceptibility to PZQ.

Six of the children sampled here in February 2005 were successfully resampled 6 months later in August 2005, with 3 days of double KKs repeated. Three children were KK-negative for *S. mansoni* (therefore, assumed to be cleared by the one additional PZQ treatment in February), and three were KK-positive. There was a significantly higher proportion of dumbbell (*t* = 5.588, df = 10, *P* < 0.001) and immobile miracidia (*t* = 2.56, df = 10, *P* = 0.027) from the individuals who were subsequently KK-negative 6 months later compared with those that were KK-positive (Figure 4).

#### Previous PZQ treatments.

There was a non-significant effect of the total number of PZQ treatments received by the children on the proportion of miracidial inactivity and shape change (ANOVA: F = 2.768, *P* = 0.063); however, this was not linearly associated with the number of treatments as predicted (regression: F = 0.213, *P* = 0.648). Instead, miracidia from individuals that had received one or three PZQ treatments (of which seven of eight of the tests were carried out pre-retreatment) had a significantly higher proportion of immobile (*t* = 3.417, df = 28, *P* = 0.002) and dumbbell miracidia (*t* = 2.677, df = 28, *P* = 0.012) than those from individuals that had received two or four PZQ treatments (where 5/7 were tested 1 week post-treatment).

## Discussion

*In vitro* PZQ has been shown to significantly affect the shape and mobility of *S. mansoni* miracidia, with responses depending on the parasites' genotype and *in vivo* PZQ exposure. Furthermore, this bioassay has been shown here to be sufficiently sensitive to serve as a predictor of clearance trends in the field, strongly endorsing the possible use of this rapid and inexpensive diagnostic test in natural foci.

Human infections that seemed to be more susceptible to PZQ (with lower intensities 1 week post-retreatment) had more dumbbell and inactive miracidia pre-retreatment than those that were less susceptible to PZQ (identified as being uncleared by an additional PZQ administration). In addition, the miracidia hatched 1 week post-retreatment also had less shape change and inactivity than those hatched before retreatment. This strongly supported our laboratory findings; more inactive miracidia were observed as parasite susceptibility increased from R to RS to S as well as miracidia from PZQ-treated mice took longer to change shape than those from untreated mice. Time to shape change may, therefore, also be an important indicator of reduced susceptibility to PZQ as well as the proportions that change shape. However, not R, as may be predicted, but RS miracidia took the longest to become immobile, although this could be explained by an overall increase in fitness in this coinfected line by the unavoidable outbreeding between lines maintained within the laboratory for many generations. Variations found in the RS group could also depend on the inheritance of PZQ resistance^30^ and potentially, the higher susceptibility of male than female schistosomes commonly reported in adult worms;^24^,^31^ this was also more recently shown in the cercarial larval stage of *S. mansoni*,^32^ and, if also common in miracidia, these results may, therefore, also depend on the ratio of male to female eggs and subsequent miracidia.

Further evidence for this test's sensitivity on miracidia from human infections was illustrated by its ability to detect differences between new infections in children who were negative 5 months previously with parasite populations assumed to be PZQ-naïve and infections in those children that may have remained infected even after two or more PZQ treatments. In addition, miracidia hatched from individuals that were KK-negative 6 months later (whose infections have, therefore, been cleared by a single subsequent treatment and classified as PZQ-susceptible) showed significantly higher levels of inactivity than those that were potentially PZQ-resistant, shown by positive KK smears 6 months after additional PZQ treatments. It may, however, be argued that these infections could have been caused by reexposure as well as some non-clearance, an explanation supported by molecular analysis of miracidia from these populations (Lamberton PHL and others, unpublished data). Although untreated groups were not investigated for ethical considerations, it can be argued that observed differences are primarily caused by PZQ exposure, particularly because of the strong significance of the paired tests where miracidia were hatched from the same individuals at two different time points.

Although efforts were made to use samples from children with a wide range of infection intensities, variations in hatching success may also explain differences in results between the two time points. However, the clear significant differences between samples hatched at the same time point from similar infection intensities that then differed greatly in their clearance after another PZQ treatment indicate that the observed differences are caused by actual variations in PZQ sensitivity in the parasites.

The effect of recent PZQ treatment seemed to be more important than the total number of treatments that an individual had received. One potential explanation may be that parasites with a higher survival to recent PZQ treatment could be assumed to be more resistant than those that have a lower survival, irrelevant of the number of previous PZQ exposures, potentially explained by relatively high levels of reinfection. An alternative explanation could be the effect of PZQ on *S. mansoni* eggs still within the host^33^ rather than on the adult worms. Mature eggs have been observed to be more susceptible to PZQ than immature eggs.^34^ Knowledge of the average time that eggs take to mature and be excreted from the body and the ability of adult worms to produce eggs under PZQ stress would help to elucidate possible causes for these differences in miracidial responses. If eggs are excreted within 1 week of production, then differences in the miracidia pre- and 1 week post-PZQ would be caused by variation in adult worm survival and their subsequent egg production. If, however, excretion takes over 1 week, then the differences observed here could be because of *in vivo* PZQ exposure directly on the eggs rather than the adults. Studies on the effect of the immune system as well as PZQ directly on the eggs may help elucidate this further and expand on the current research on adult worm teguments and the mode of action of PZQ.^35^ Detailed *in vitro* research can be hugely helpful in not only monitoring PZQ resistance but also understanding the drug's mechanisms.

The results from this study show that the proportion of dumbbell-shaped miracidia and inactivity are strong indicators of reduced sensitivity to PZQ within human infections. The number of normal-shaped miracidia, however, actually seemed to be higher in samples hatched pre-retreatment than 1 week later. Miracidia that remain normal under *in vitro* PZQ pressure are considered to be resistant. Because this study was carried out 6 months after an MDA intervention, the presence of more normal-shaped miracidia pre-retreatment could be explained by parasites that are slightly resistant to PZQ and were uncleared by the last PZQ dose 6 months ago but were now cleared by an additional PZQ treatment, especially as the proportion of normal miracidia pre-PZQ were only weakly significantly higher in the paired samples. Such results may also, however, be partly caused by underlying natural parasite genetic heterogeneity,^36^ supporting the finding that no significant difference was found in the larger group of unpaired samples.

For modeling evolutionary predictions on the spread of certain genotypes, such as PZQ resistance, throughout a population, key factors involve knowing how many resistant or tolerant parasites there are in an untreated population. If one population, for example, is more susceptible than another population, then the more susceptible population will have fewer genes for resistance in an unselected group and therefore, be less likely to develop resistance after chemotherapy. It is, therefore, of vital importance that more research be carried out in true PZQ-naïve populations, particularly involving methods such as those discussed here, to analyze the proportion and later, any spread of reduced sensitivity to PZQ. In addition, investigations under contrasting MDA conditions within and between different countries for *S. mansoni* as well as studies on *Schistosoma haematobium*, which seems to be more susceptible to PZQ than *S. mansoni*,^37^ would provide vital information on the status and understanding of parasite PZQ susceptibility. These would also help to clarify standard operating procedures needed for such a test so that it could be easily and accurately incorporated within an MDA program.

## Conclusions

The impact of *in vitro* PZQ on miracidial shape and mobility was shown to closely reflect *in vivo* susceptibility in laboratory untreated mice; however, even S parasites that survived *in vivo* PZQ were less susceptible *in vitro*. PZQ miracidial test results were uniquely shown to be associated with clearance trends in individual human infections, indicating a highly feasible use of *in vitro* PZQ as a diagnostic tool to detect potential resistant genotypes and provide important insights into parasite population structure within this and similar schistosome-endemic regions. Although clearance rates remain high in these MDA areas, this study has shown, for the first time, that miracidia populations hatched after PZQ treatment are phenotypically different from those hatched before and that those hatched before subsequent clearance are different from those that remained uncleared. Our results thereby illustrate the exciting possible uses of this bioassay as well as highlight the need for continued monitoring of the parasite populations, potentially through such assays, as treatments progress. Such a phenotypic measure for testing potential resistance may be highly important now with the increase in and expansion of MDA, particularly given the current lack of genetic markers available.
